# Supplementary material for: Telomere Dynamics in the Diploid and Triploid Rainbow Trout (Oncorhynchus mykiss) Assessed by Q-FISH Analysis
Source: Genes (Basel). 2020 Jul 13;11(7):786. doi: 10.3390/genes11070786 (PMC7397301; doi:10.3390/genes11070786)
Supplement: Supplementary file 1 [file genes-11-00786-s001.pdf]

symbol od examined fish	Stage of development	Ploidy	Fluorescence (auf)
1de	Embryo	2N	25,25
2de	Embryo	2N	29,5
3de	Embryo	2N	19,25
4de	Embryo	2N	19,65
5de	Embryo	2N	27,35
6de	Embryo	2N	23,2
7de	Embryo	2N	15,7
8de	Embryo	2N	39,65
1te	Embryo	3N	26,8
2te	Embryo	3N	45,25
3te	Embryo	3N	21,5
4te	Embryo	3N	41,6
5te	Embryo	3N	24,3
6te	Embryo	3N	39,5
7te	Embryo	3N	21,5
8te	Embryo	3N	25,25
1ld	Larva	2N	25,45
2ld	Larva	2N	26,4
3ld	Larva	2N	27,75
4ld	Larva	2N	26,85
5ld	Larva	2N	23,25
6ld	Larva	2N	23,5
7ld	Larva	2N	33,35
8ld	Larva	2N	26,35
1lt	Larva	3N	21,95
2lt	Larva	3N	21,5
3lt	Larva	3N	35,25
4lt	Larva	3N	30,15
5lt	Larva	3N	24,95
6lt	Larva	3N	27,9
7lt	Larva	3N	27,75
8lt	Larva	3N	45,6
1dj	1 year old	2N	32,85
2dj	1 year old	2N	13,7
3dj	1 year old	2N	26,5
4dj	1 year old	2N	15,6
5dj	1 year old	2N	20
1tj	1 year old	3N	24,3
2tj	1 year old	3N	17,35
3tj	1 year old	3N	19,35
4tj	1 year old	3N	19,4
5tj	1 year old	3N	12,25
1ds	2 years old	2N	16,7
2ds	2 years old	2N	11,25
3ds	2 years old	2N	12,4
4ds	2 years old	2N	11,3
5ds	2 years old	2N	13,55
6ds	2 years old	2N	10,2
1ts	2 years old	3N	11,5

2ts	2 years old	3N	8,4
3ts	2 years old	3N	10,85
4ts	2 years old	3N	12,55
5ts	2 years old	3N	12,75
6ts	2 years old	3N	12,2
1da	3 years old	2N	44,1
2da	3 years old	2N	32,4
3da	3 years old	2N	69,75
4da	3 years old	2N	26,1
5da	3 years old	2N	13,55
1ta	3 years old	3N	12,65
2ta	3 years old	3N	25,05
3ta	3 years old	3N	18,55
4ta	3 years old	3N	17,85
5ta	3 years old	3N	16,55

sd
6,8
9,55
3,2
5,65
7,45
3,2
1,9
10,8
6,7
11,5
3,5
11
5,85
9
4,35
5,1
5,1
5,75
6,3
3,7
3,15
4,85
6,1
4,35
5,3
5,6
7,3
6,15
5,4
6,2
3,85
9
9,25
2,5
7,25
2,15
4,15
4,55
3,1
2,75
2,8
1,75
2,5
1,3
1,4
1,65
2,15
2,3
3

0,95
2,05
1,55
2,3
1,95
15,35
6,75
7,7
5,1
1,65
1,95
7,5
4,2
5,45
1,65
